# Proteomics Analyses Reveal Functional Differences between Exosomes of Mesenchymal Stem Cells Derived from The Umbilical Cord and Those Derived from The Adipose Tissue 

**DOI:** 10.22074/cellj.2021.6969

**Published:** 2021-03-01

**Authors:** Bin Liu, Guanglei Qiao, Wen Cao, Chenlu Li, Shaojun Pan, Lirui Wang, Yanlei Liu, Lijun Ma, Daxiang Cui

**Affiliations:** 1. Institute of Nano Biomedicine and Engineering, Shanghai Engineering Research Center for Intelligent Diagnosis and Treatment Instrument, Department of Instrument Science and Engineering, School of Electronic Information and Electrical Engineering, Shanghai Jiao Tong University, China; 2. National Center for Translational Medicine, Collaborative Innovational Center for System Biology, Shanghai Jiao Tong University, China; 3. Department of Oncology, Tongren Hospital, Shanghai Jiao Tong University School of Medicine, China; 4. Bio-X Institutes, Key Laboratory for the Genetics of Developmental and Neuropsychiatric Disorders (Ministry of Education), Shanghai Jiao Tong University, China; 5. School of Laboratory Medicine and Life Science, Wenzhou Medical University, University Town, Chashan, China; 6. School of Biomedical Engineering, Shanghai Jiao Tong University, China

**Keywords:** Complement and Coagulation Cascades, Exosomes, Mesenchymal Stem Cells, Proteomics Analysis

## Abstract

**Objective:**

We aimed to identify the differentially expressed proteins (DEPs) and functional differences between exosomes derived from mesenchymal stem cells (MSCs) derived from umbilical cord (UC) or adipose tissue (AD).

**Materials and Methods:**

In this experimental study, the UC and AD were isolated from healthy volunteers. Then, exosomes from UC-MSCs and AD-MSCs were isolated and characterized. Next, the protein compositions of the exosomes were examined via liquid chromatography tandem mass spectrometry (LC-MS/MS), followed by evaluation of the DEPs between UC-MSC and AD-MSC–derived exosomes. Finally, functional enrichment analysis was performed.

**Results:**

One hundred and ninety-eight key DEPs were identified, among which, albumin (ALB), alpha-II-spectrin (SPTAN1), and Ras-related C3 botulinum toxin substrate 2 (RAC2) were the three hub proteins present at the highest levels in the protein-protein interaction network that was generated based on the shared DEPs. The DEPs were mainly enriched in gene ontology (GO) items associated with immunity, complement activation, and protein activation cascade regulation corresponding to 24 pathways, of which complement and coagulation cascades as well as platelet activation pathways were the most significant.

**Conclusion:**

The different functions of AD- and UC-MSC exosomes in clinical applications may be related to the differences in their immunomodulatory activities.

## Introduction

Mesenchymal stem cells (MSCs) are pluripotent stem cells with the abilities of self-renewing and differentiating into various cell types, such as osteoblasts, chondrocytes, myocytes, and adipocytes, under appropriate conditions (1). MSCs have specific immune properties that allow them to survive in a heterogeneous environment (2). Umbilical cordderived MSCs (UC-MSCs) are the most primitive MSCs, and they have been proven to be effective in disease therapy, such as in lupus erythematosus (3), psoriasis (4), and rheumatoid arthritis (5). Adipose tissue-derived mesenchymal stem cells (AD-MSCs), which can differentiate into osteoblasts, chondroblasts, adipose precursor cells, and cardiomyocytes, hold great promise for use in wound healing and treating kidney injuries (6, 7). Although the clinical applications of UC-MSCs and AD-MSCs are extensive, their applications are somewhat different. Additionally, there are various limitations for the clinical applications of MSCs on the whole. For instance, except for UC-MSCs, MSC collection procedures are invasive and laborious. Furthermore, proliferation and differentiation abilities of MSCs decrease in culture after several passages (8, 9). In spite of the function of MSCs that has been confirmed in regenerative medicine and immunomodulatory diseases, the wide application of MSCs is restricted owing to the limitations in their source and stability (8).

Exosomes are small bilayer vesicles of 30-100 nm in diameter that are released from cells. They are involved in intercellular communication by transferring cellular components between cells (10). Many studies have shown that MSC-derived exosomes have functions similar to those of MSCs, such as immune regulation and promoting regeneration of damaged tissues (11). Relative to MSCs, exosomes are more stable and retainable in the host following their allogeneic administration due to a lower host-versusgraft reaction (10). Exosomes derived from MSCs may provide an alternative therapy for various diseases, especially for degenerative diseases. Li et al. (12) have reported that transplantation of exosomes derived from UC-MSCs alleviates liver fibrosis induced by CCl_4_. Similarly, exosomes from UC-MSCs repair cisplatin-induced acute kidney injury and acute myocardial ischemic injury (13, 14). In addition, in a previous study exosomes derived from UC-MSCs showed immunomodulatory effects on* in vitro* stimulated T cells and promoted cell migration in a breast cancer cell model through the Wnt signaling pathway (15, 16). Combination of ADMSCs- and AD-MSC-derived exosomes also significantly reduced the brain infarct volume in strove rats, and protected the kidneys from acute ischemia-reperfusion injury (17, 18).

Exosomes usually contain lipids, miRNAs, mRNAs, and proteins that can recognize their target cells and modulate their functions. In recent years, exosome proteomes have been at the center of attention in biomedical research. Thousands of proteins have been found in exosomes, several hundreds of which are common in at least two sets, and only two proteins are shared by more than four sets (11). It is well known that exosomes are membranous vesicles released by cells, which exist in blood, breast milk, saliva, malignant effusions, and also in the supernatants of cell cultures (20). In recent years, exosomes derived from MSCs have been applied in the clinics. As proteins mediate most of the intracellular physiological processes and communication between cells, mass spectrometry proteomics methods and proteomics have been widely used in elucidating biological processes (21). In the present study, exosomes from UC-MSCs and AD-MSCs were isolated and their protein compositions were examined by liquid chromatography-tandem mass spectrometry (LC-MS/MS). Then, functional enrichment analysis of the differentially expressed proteins (DEPs) between the two exosomes was performed. The purpose of this research was to evaluate the DEPs and potential functional differences between the exosomes derived from UC-MSCs and those derived from AD-MSCs. 

## Materials and Methods

### Materials

In this experimental study, MSCs serum-free medium was ordered from Shanghai Pumao Biotechnology (Shanghai, China), and trypsin and Dulbecco’s phosphate buffered saline (DPBS) were ordered from Gibco (Grand Island, NY, USA). The antibodies of APC-anti-human CD73, FITC-anti-human CD90, PerCP-Cy5.5- antihuman CD105, PE-anti-human CD34, PE-anti-human CD45, and PE-anti-human HLA-DR were purchased from BD Pharmingen (NJ, USA). The CD63 and β-actin antibodies were ordered from Absin Bioscience (Shanghai, China) and Cell Signaling Technology (Danvers, MA, USA), respectively. 

### Cell culture and supernatant collection

The collection and use of all human umbilical cords or adipose tissues from healthy volunteers in this study was approved by Ethics Committee of Shanghai Tongren Hospital (No.201501801). UC-MSCs were acquired by through direct explant method using umbilical cords donated by healthy women immediately after giving birth (22). ADMSCs were acquired from the adipose tissues of healthy volunteers using the enzymatic digestion method (23). All the donors provided their informed consents prior to tissue donation. Cellular morphologies of the UC- and AC-MSCs were examined and photographed using a light microscope (Nikon TS100). The UC- and AD-MSCs were plated at the density of 8000 cells/cm2 in T175 flasks for 3-5 passages in serum-free medium. The supernatants of the cultures were collected after 72 hours for exosome collection. The total supernatant volume collected for each cell type was 50 mL.

### Characterization of the surface markers on UC-MSCs and AD-MSCs

Surface marker characterization was performed by flow cytometry. Briefly, the UC-MSCs and AD-MSCs were detached with trypsin and washed with DPBS. Afterward, APC-anti-human CD73, FITC-anti-human CD90, PerCPCy5.5-anti-human CD105, PE-anti-human CD34, PEanti-human CD45, and PE-anti-human HLA-DR were added into the tubes containing 5-10×10^5^ cells. After twenty minutes of incubation in the dark. the cells were washed with DPBS and processed with FACS Calibur system (BD Bioscience, USA). 

### Extraction and identification of exosomes

To remove the residual cells and fragments, the supernatants of both cell types were consecutively centrifuged at 800 g for ten minutes and 12,010 g for twenty minutes at 4˚C. Then the samples were centrifuged at 100,010 g for two hours at 4˚C. After washing, the precipitates were resuspended in 100 μL DPBS. Finally, the exosomes were stored at -80˚C for future usage.

The morphologies of the exosomes were examined using a transmission electron microscope (TEM) JEM 2100F (JEOL, Japan). The particle size distribution of the exosomes was assessed by the particle analysis system: qNano (Izon science, Oxford, UK). The expression level of CD63 on the exosomes was evaluated by the BCA Protein Assay Kit (Sangon Biotech, Shanghai, China) and western blotting assay.

### Mass spectrometry analysis

Mass spectrometry analysis Exosome-associated proteins were studied using LCMS/MS. Briefly, the supernatant and exosome solutions of UC- and AD-MSCs were all sonicated before the protein contents were extracted. Afterward, 1M dithiothreitol and 1 M iodoacetamide were included to reduce and alkylate the extracted proteins, respectively. The samples were then digested using 20 ng/μL trypsin overnight at 37˚C. Next, the mixtures were centrifuged at 12,010 rpm for twenty minutes. Then the filtrate was collected and dried at 55˚C to obtain the polypeptides. The dried polypeptide samples were reconstituted in 0.1% aqueous formic acid, followed by desalting via ZipTip C18 columns (Thermo Scientific, Waltham, MA, USA). The samples were recovered from the columns with water containing 2% acetonitrile and 0.1% formic acid. Finally, the samples were analyzed with Nano Liquid Chromatography–Orbitrap Mass Spectrometry (Easy-nLC1200, Q-Exactive Plus, ThermoFisher Scientific). In the present study, 16 samples were analyzed, comprising 4 replicates from each supernatant and exosome sample derived from UC-MSCs or AD-MSCs (Sup UC, Sup AD, Exosome UC, and Exosome AD).

For the mass spectrometric analysis, an electrospray positive ion source and secondary data-dependent acquisition (data-dependent acquisition, DDA) mode (Target SIMdd MS2) were used. The parameters of full mass were as follows: 2.0 KV spray voltage, 320˚C capillary temperature, 350-1500 m/z scan range, 70,000 resolution, 3e6 AGC target, and 50 ms maximum ion injection time (MIT). The parameters of dd-MS2/dd-SIM were as follows: 20-2000 m/z scan range, 17,500 resolution, 1e5 AGC target, 45 ms MIT, and 1.6 m/z isolation window. The most abundant 20 peptides were subjected to a secondary fragmentation using high energy collisional dissociation (loop count=20, MSX count=1, TopN=20). The normalized collision energy (NCE) was 28. Likewise, the ions with a charge number of 1 and those≥8 were excluded. The dynamic exclusion time was 30 seconds.

### Data preprocessing

Data were obtained by Nano-ESI-LC-MS/MS by searching the Sequest HT engine, and the proteins of each group were identified by the Proteome Discoverer software (2.2.0.388, ThermoFisher Scientific). Protein analysis was performed using UniProt analysis software (https://www.uniprot.org/) with fixed carbamidomethylcysteine, variable methionine-oxidation asparagine/ glutamine deamidation, and N-terminal acetyl that was allowed against the complete human proteome. The precursor mass tolerance was 10 ppm and the fragment mass tolerance was 0.02 Da, with the maximum missed cut site allowed being 2. The cut-off value for false discovery rate (FDR) was ≤1%.

### Identification of the differentially expressed proteins 

The log fold change (log FC) was calculated based on the total abundance ratio and the P value. The DEPs between Exosome UC vs. Exosome AD and Sup UC vs. Sup AD were selected with the P value<0.05 and |log2FC| >0.585 (1.5 fold) as the threshold of DEPs. In addition, the volcano and Veen maps were constructed. 

### Protein-protein interaction network of the differentially expressed proteins 

Search tool for the retrieval of interacting genes/proteins (STRING) database was applied to analyze the protein-protein interaction (PPI) of the DEPs (23). The PPI relationship pairs, of which the threshold Required Confidence >0.4 were used in the formation of the PPI network by Cytoscape (http://www.cytoscape.org/). Moreover, CytoNCA plugin (Version 2.1.6) was selected to study the topological features of the nodes in the PPI network with the "without weight" parameter. Based on the order of the degree centrality (DC), betweenness centrality (BC), and closeness centrality (CC) of the nodes, the top 10 key nodes (also named hub proteins) were identified. 

### Functional enrichment analysis of the DEPs

The clusterProfiler of R package (Version 3.2.11) was used to perform the KEGG pathway and gene ontology (GO) enrichment analyses. P values <0.05 were considered significant, and the top 20 of the GO and pathway items were taken into account in the analysis. ClueGO and CluePedia plugins of cytoscape were used for functional clustering analysis of the target proteins. The network map of all target functional proteins was constructed by cytoscape. In the ClueGO plugin, the value of kappa showed the relationship between the GO terms according to the overlapping genes. Meanwhile, GO functions were grouped according to the kappa coefficients. A high kappa coefficient indicates a stronger correlation between the GO items. 

### Mapping the key pathway

Key pathways were mapped by the KEGG MAPPER, and the key proteins were marked.

## Results

### Cell surface markers of MSCs derived from UC and AD

**Fig.1:**
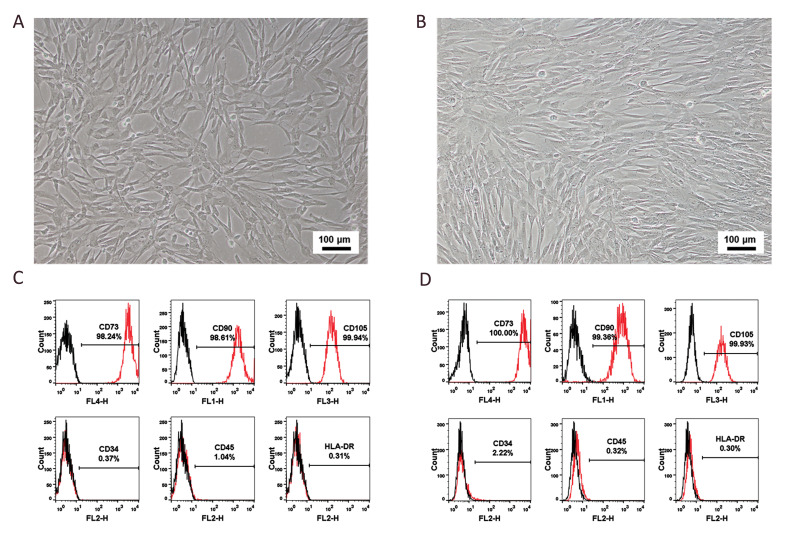
The morphology and surface biomarkers of UC- and AD- MSCs. **A** and** B. **show the morphologies of UC-and AD-MSCs with a magnification of 10X. **C** and **D.** show the surface markers of UC- and AD-MSCs. AD; Adipose tissue, UC; Umbilical cord, and MSCS; Mesenchymal stem cells.

UC-MSCs and AD-MSCs were characterized based on the International Society for Cellular Therapy (ISCT) minimal definition criteria flow cytometry (24). In culture, UC-MSCs had a long fusiform morphology with vortexlike adherent projections. On the other hand, although AD-MSCs were also fusiform, they grew in parallel to each other or in a spiral pattern (Fig .1A, B). In addition, the majority of both UC- and AD-MSCs expressed CD73 (98.24 and 100.00%, respectively), CD90 (98.61 and 99.36%), and CD105 (99.94 and 99.93%) (Fig .1C, D). In contrast, only a few UC-MSCs and AD-MSCs were positive for CD34 (0.37 and 2.22%, respectively), CD45 (1.04 and 0.32%), and HLA –DR (0.31 and 0.30%). All the results indicated that the cell morphology and surface markers of the UC- and AD-MSCs were in agreement with the ISCT standards.

### Morphological characterization of the exosomes

**Fig.2:**
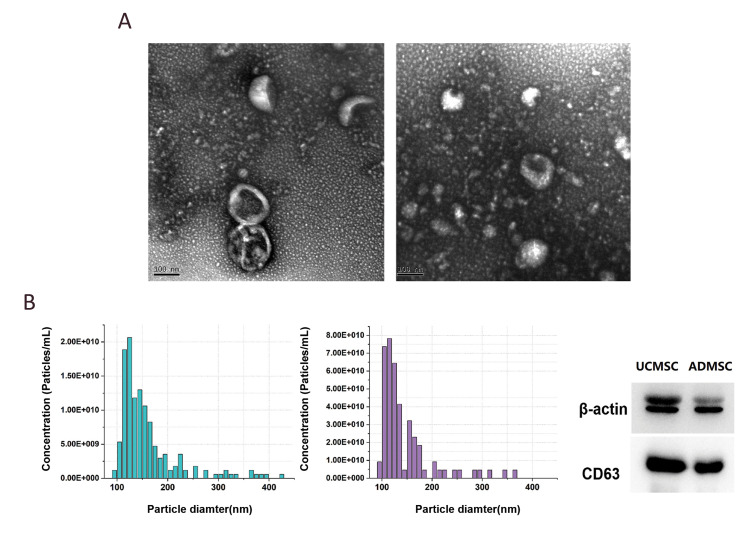
Sizes and protein characteristics of exosomes. **A.** The TEM images of exosomes derived from UC- and AD- MSCs. **B.** The particle size distribution of exosomes derived from UC- and AD- MSCs. **C. **Western blot results of exosomes from UC- and AD- MSCs. AD; Adipose tissue, UC; Umbilical cord, and MSCS; Mesenchymal stem cells.

The exosome morphologies were examined by TEM. The vesicles observed had a well-defined circular structure with a lipid bilayer membrane and a diameter of approximately 100 nm (Fig .2A). There was no obvious morphological difference between the exosomes derived from UC-MSCs and ADMSCs. The results of particle size distribution (Fig .2B) were consistent with the TEM results. In addition, the membrane-specific protein CD63 (28-29 kDa) was detected on the vesicles (Fig .2C). These results confirmed successful isolation of the exosomes.

### DEPs between exosome UC and exosome AD, and between Sup UC and Sup AD

**Fig.3:**
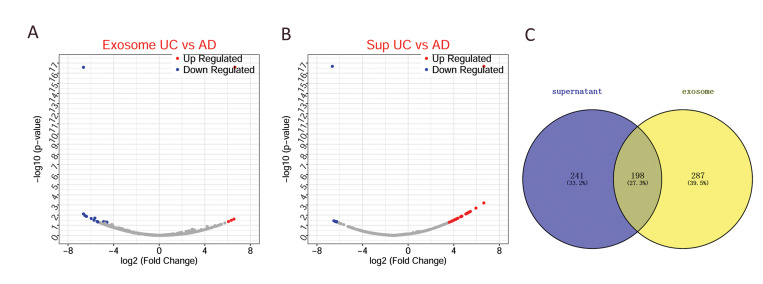
DEPs between exosomes derived from the UC- and AD- MSCs. **A.** The volcano figure of the DEPs between exosomes from the UC- and AD- MSCs. **B.** The volcano figure of the DEPs between supernatant from the UC- and AD- MSCs. **C.** Venn diagram of the DEPs. Blue and red dots represent downregulated and upregulated proteins, respectively. The gray color represents the normally regulated proteins, abscissa is log2 FC, and ordinate is log_10_P. AD; Adipose tissue, UC; Umbilical cord, DEPs; Differentially expressed proteins, and MSCS; Mesenchymal stem cells.

There were 458 DEPs between Exosome UC and Exosome AD comprising 430 upregulated and 55 downregulated proteins (Fig .3A, B). Furthermore, 439 DEPs were identified between Sup UC and Sup AD, comprising 296 upregulated and 143 downregulated proteins. There were 198 members in the intersection of the DEPs between exosome UC and exosome AD, and between Sup UC and Sup AD (Fig .3C), of which 163 DEPs were synergistically expressed proteins. Exosomes were present in the supernatants in sufficient quantities to assess for functional differences between the two exosome types by studying the intersection of the two DEPs.

### Protein-protein interaction network analysis

**Fig.4:**
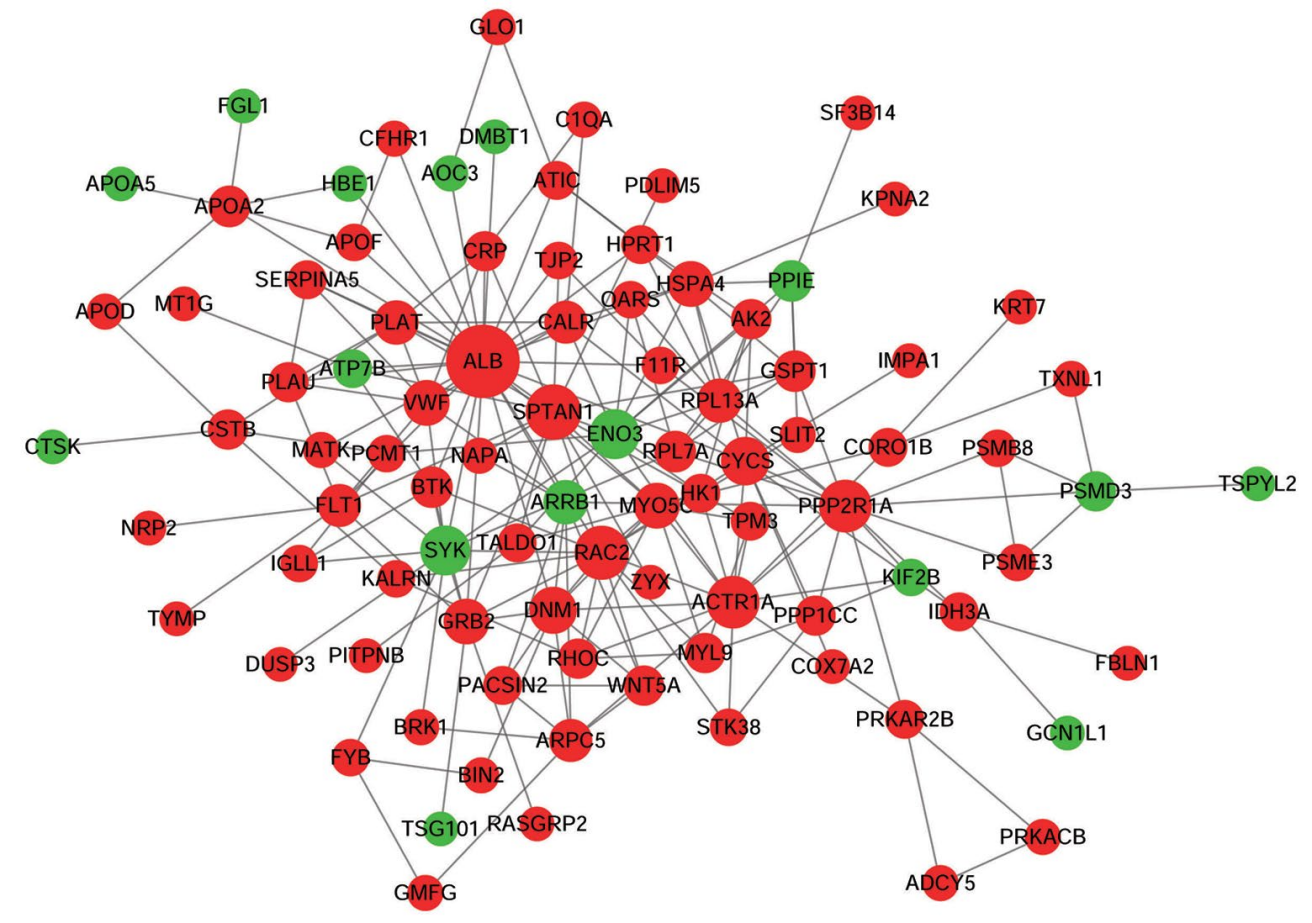
The PPI network based on 198 shared DEPs. The green and red colors represent downregulated and upregulated proteins, respectively. The dot size represents the degree of connectivity, as the higher the degree of connectivity, the larger the dot. DEPs; Differentially expressed proteins, and PPI; Protein-Protein interaction.

The PPI network was formed based on the 198 common DEPs mentioned above. This study found that the PPI network contained 92 nodes and 210 interaction pairs (Fig .4). The topological property analysis of the nodes revealed that albumin (ALB), alpha-II-spectrin (SPTAN1), and Ras-related C3 botulinum toxin substrate 2 (RAC2) had higher scores compared to other proteins. Based on the topological property analysis, the top 5 proteins (also named hub proteins) of the PPI network were ALB, SPTAN1, RAC2, protein phosphatase 2, regulatory subunit A (PPP2R1A), and alpha-centractin (ACTR1A).

### Functional enrichment analysis

**Fig.5:**
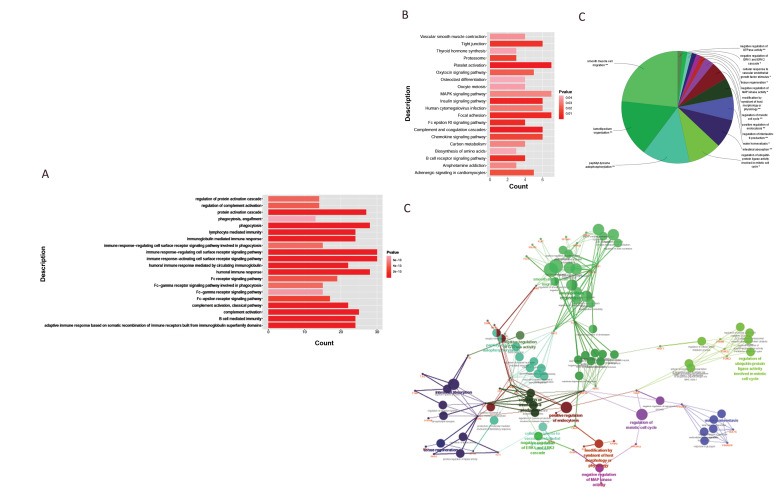
The functional enrichment analyses of the DEPs. **A.** The histogram of GO enrichment analysis based on the DEPs.** B.** KEGG pathway of DEPs. The vertical axis in A and B is the functional item and the horizontal axis is the count, which is the number of enriched proteins. The deeper the red color, the smaller the P value, indicating a higher significance. **C.** The Pie chart of functional classification. Different colors represent different types of functions, and the labels are representative function items. **D.** The network of ClueGO function. The red label is the target protein and the other dots are GO functional terms. The two-point line represents the correlation between different functions. Different colors represent different groups, and the smaller the P value, the larger the dot. Meanwhile the larger the kappa coefficient, the thicker the line. DEPs; Differentially expressed proteins, Go; Gene ontology, and KEGG; kyoto encyclopedia of genes and genomes.

A total of 438 GO items were obtained based on the functional enrichment analysis, which was performed on the 163 synergistically expressed DEPs. The DEPs were mainly enriched in the GO items associated with immunity, complement activation, and protein activation cascade regulation (Fig .5A). Additionally, the DEPs were enriched in 24 pathways, including those associated with insulin signaling, focal adhesion, complement and coagulation cascades, and platelet activation (Fig .5B). Among these, the complement and coagulation cascade pathway and platelet activation were the most significant pathways in the whole 24 pathways. 

Functional clustering analysis performed by the ClueDO plugin revealed that the enriched functions were divided into 15 categories. Figure 5C shows the representative functional items of each category, such as smooth muscle cell migration, lamellipodium organization, peptidyl tyrosine autophosphorylation, negative regulation of GTPase activity, and intestinal absorption (denoted by **, if the term/group was greater than significant, P value<0.001). In addition, correlation analysis was performed on the functional categories to obtain a functional clustering network (Fig .5D). The colors in Figures 5 C and D correspond to each other, and the same color represents the same type of GO function. The number of GO terms connected to the protein nodes (red markers) and the thickness of the links indicates the significance of each protein. Microfilament-associated proteins BRK1, recombinant rabbit coronin-1B (CORO1B), slit homolog 2 protein N-product (SLIT2), apolipoprotein A-II precursor (APOA2), apolipoprotein A-5 precursor (APOA5), and β-arrestin-1 (ARRB1) were more significant than others. Figure 5D also shows the correlation between the GO items—the smaller the P value, the larger the dot, indicating a stronger correlation. It is clear from these results that intestinal absorption, positive regulation of endocytosis, regulation of the meiotic cell cycle, and smooth muscle cell migration are processes where protein function is substantial.

### Mapping the key pathway

**Fig.6:**
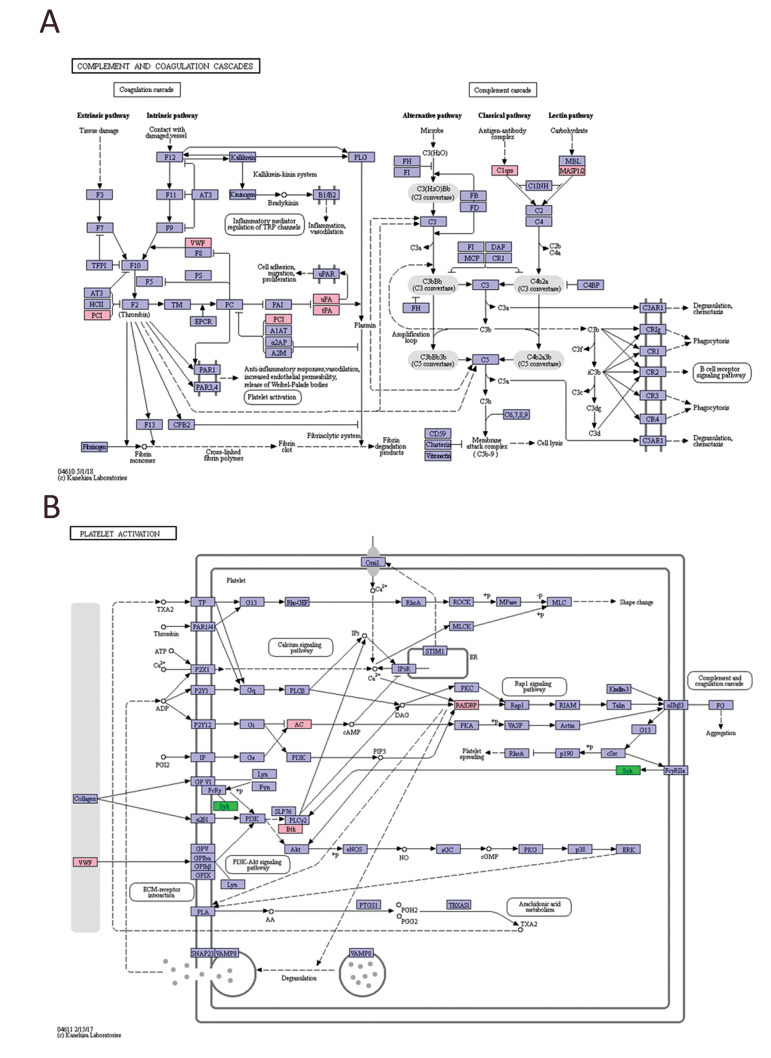
The regulatory network of different pathways. **A.** The regulatory network of the complement and coagulation cascade pathway. **B.** The regulatory network of the platelet activation pathway. Pink represents the differential protein that is upregulated in the pathway, and green is the downregulated protein in UC-MSC exosomes compared with AD-MSC exosomes. AD-MSCS; Adipose mesenchymal stem cells, and UC; Umbilical cord.

To further study the function of exosomes, the potential regulatory networks of the key pathways, the complement and coagulation cascade and platelet activation, were mapped. Figure 6A shows the complement and coagulation cascade pathway, which contains both extrinsically and intrinsically regulated cascades. When compared with the Exosome AD, the expressions of protein C inhibitor (PCI), von Willebrand Factor (vWF), urokinase type plasminogen activator (uPA), plasminogen activator (tPA), complement regulatory protein C1qrs, and human mannan-binding lectin-associated serine protease 1/2 (MASP1/2) in the Exosome UC were significantly upregulated. Figure 6B shows the platelet activation pathway, which has a significant association with the complement and coagulation cascade pathway. Adenylate cyclase (AC), Rasguanine nucleotide releasing factor (RASGRP), von willebrand factor (vWF), and tyrosine protein kinase Btk were upregulated in the Exosome UC, whereas tyrosine kinase Syk was downregulated.

## Discussion

The widespread use of MSC exosomes in the repair and regeneration of damaged tissues is closely related to immune regulation (25). According to Neerukonda et al. (26), MSC exosomes can regulate innate and adaptive immune responses during infection, inflammation, and virus-associated pathology (27). The immunoregulatory effect of MSC exosomes may be associated with suppression of T lymphocyte function, natural killer cell cytotoxic activity, and B cell activation, proliferation and secretion. It may also be associated with modulation of macrophage differentiation and dendritic cell maturation (28). Proteomic analyses have shown that MSC exosomes contain various cytokines, such as interleukin-10 (IL10), interleukin-6 (IL-6), macrophage stimulating factor (MSF), transforming growth factor β (TGF β), prostaglandin E2, platelet-derived growth factor (PDGF), vascular endothelial growth factor (VEGF), and fibroblast growth factor (FGF) among others, indicating that MSC exosomes have immunomodulatory functions (29). 

In this study, the DEPs of exosomes from UC- and AD-MSCs were mainly enriched in immune functions, suggesting that these two exosome types highly differ in immune regulation. The complement and coagulation cascades and the platelet activation pathway were the most important among the 24 pathways related with GO items. The proteins BRK1, CORO1B, SLIT2, APOA2, APOA5, and ARRB1 in the functional clustering network were considered more significant than other proteins based on the Main GOs. Taken together, these results suggest that these two exosome types may be used for different clinical applications. 

The complement cascade has serum proteins that are activated by antigen-antibody complexes, causing pathogenic microorganisms to be cleaved or phagocytosed, thereby mediating immune and inflammatory responses (30, 31). The complement system has an important roles in exosome-related biological functions. Neuronal exosomes promote synaptic pruning through upregulation of complement cascade factors (32). Moreover, astrocytederived exosomes in Alzheimer patient cells has shown high complement levels (33). In addition, the complement and coagulation cascade was the star signaling pathway in the proteomic research of exosomes. Wong et al. (34) reported that in the serum exosomes of acute ulcerative colitis mice, caused by dextran sulfate sodium, the complement and coagulation cascade pathway was activated. A proteomic profile analysis on the pathology of preeclampsia also revealed that cord exosome proteins might play an important role via the complement and coagulation cascades (34). In our study, the DEPs were obviously enriched in the complement and coagulation cascade pathway, indicating that the different functions of exosomes of AD- and UC-MSCs were related to the complement and coagulation cascades. 

Platelet activation was another significant signaling pathway in our study. It has been reported that exosomes are novel effectors of human platelet lysate activity (35). There are many studies focusing on bioactivities of exosomes derived from different platelets. For instance, exosomes from septic shock patients can induce myocardial dysfunction (36). Guo et al. (37) revealed that exosomes from plasma that contain large amounts of platelets may improve re-epithelialization of chronic skin wounds in diabetic rats. However, there are few studies on the activating effects of exosomes on platelets, especially in various types of MSCs (38). In our study, we found that the DEPs were mainly involved in platelet activation, which gave us a new perspective to assess the different functions between AD- and UC-MSC exosomes.

## Conclusion

The DEPs between AD- and UC-MSC exosomes were significantly enriched in immunity, complement system, coagulation cascade, and platelet activation pathways. The different functions of AD- and UC-MSC exosomes in clinical applications may be related to their immunomodulatory activities.
